# Supplementary material for: n-3 Polyunsaturated Fatty Acids Decrease Long-Term Diabetic Risk of Offspring of Gestational Diabetes Rats by Postponing Shortening of Hepatic Telomeres and Modulating Liver Metabolism
Source: Nutrients. 2019 Jul 23;11(7):1699. doi: 10.3390/nu11071699 (PMC6683104; doi:10.3390/nu11071699)
Supplement: Supplementary file 1 [file nutrients-11-01699-s001.pdf]

Supplementary Figure S1. Score plots of PCA and OPLS-DA of all groups and pairwise OPLS-DA between groups and corresponding permutation tests of liver when offspring grew to 11 months old. n=8 ~ 10 rats/group.

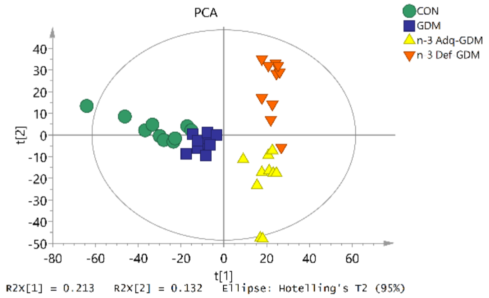

(**PCA**)

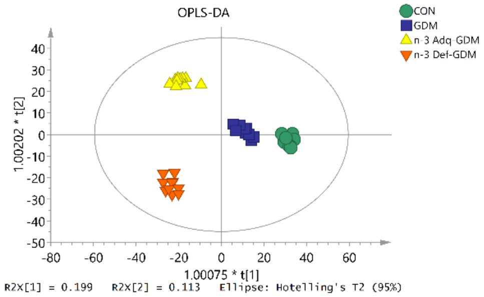

(**OPLS-DA**)

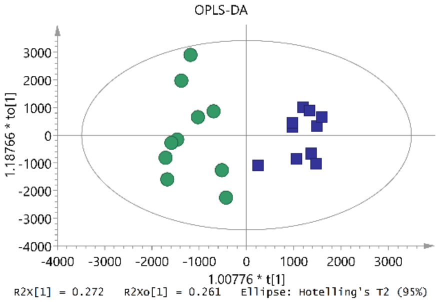

(**OPLS-DA (GDM vs. Con)**)

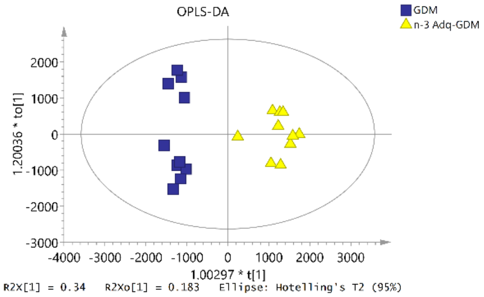

(**OPLS-DA (n-3 Adq-GDM vs. GDM)**)

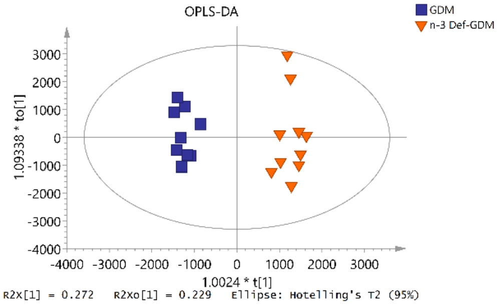

(**OPLS-DA (n-3 Def-GDM vs. GDM)**)

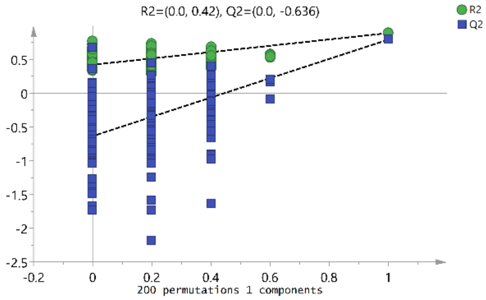

(**Permutation test (GDM vs. Con)**)

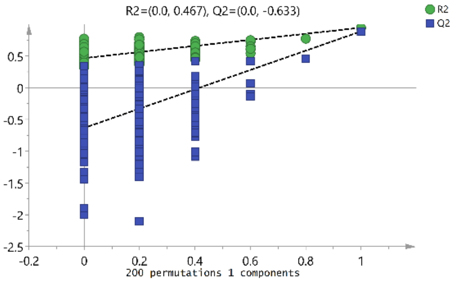

(**Permutation test (n-3 Adq-GDM vs. GDM)**)

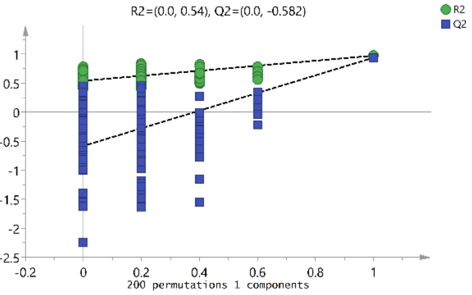

(**Permutation test (n-3 Def-GDM vs. GDM)**)

Supplementary Figure S2. Metabolic pathway enrichment maps of liver of offspring at 11 months old based on changed metabolites between GDM offspring vs Control offspring

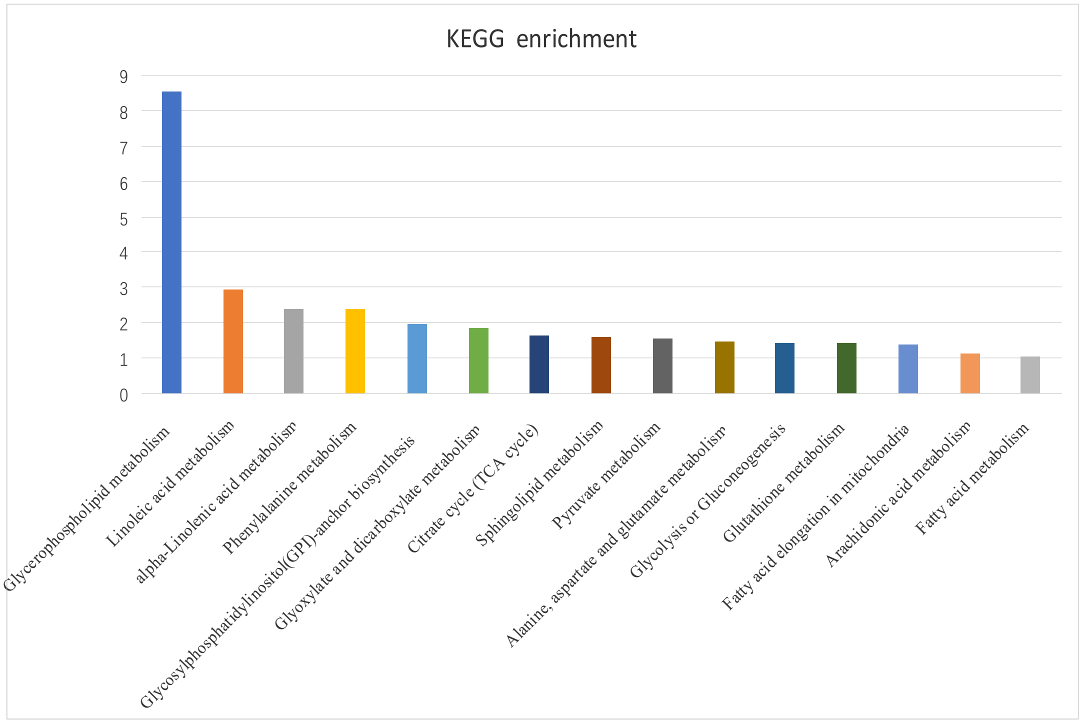

Supplementary Figure S3. Effect of n-3 PUFA on body weight of gestational diabetes mellitus (GDM) offspring. Bars are mean ±SD. n=8 ~ 12 offspring rats/group. *P<0.05, **P<0.01, vs Control offspring (Con); #P<0.05, vs GDM offspring (GDM); $P<0.05, vs n-3 Adequate-GDM offspring (n-3 Adq-GDM).

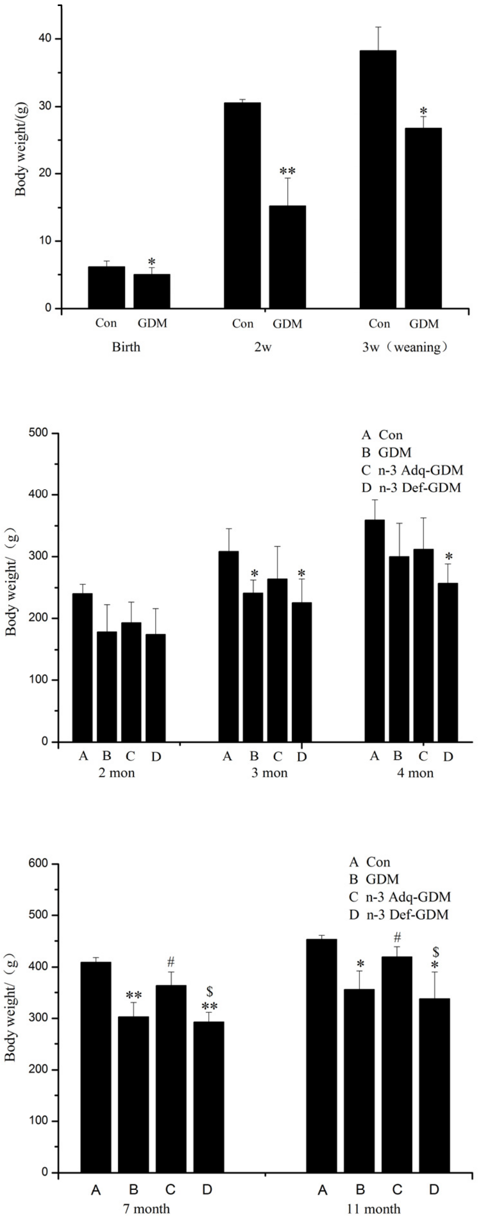

Supplementary Figure S4. Effect of n-3 PUFA on TG and TC in liver of gestational diabetes mellitus (GDM) offspring. (A) TG; (B) TC. Bars are mean ±SD. n=8 ~ 10 offspring rats/group. $^{*}P<0.05$, $^{**}P<0.01$, vs Control offspring (Con); $^{\#}P<0.05$, $^{\#\#}P<0.01$, vs GDM offspring (GDM); $^{\$\$}P<0.01$, vs n-3 Adequate-GDM offspring (n-3 Adq-GDM).

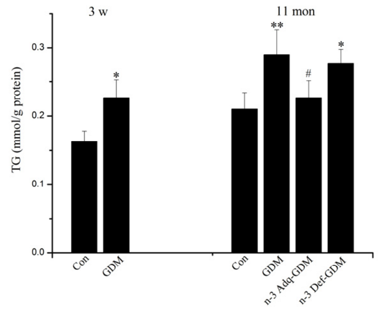

(A)

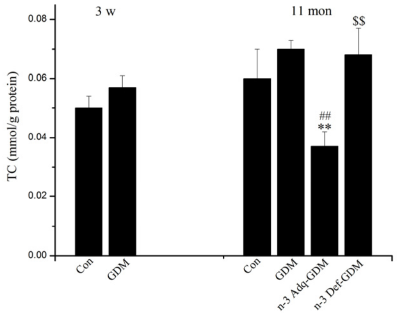

(**B**)

Supplementary Table S1. R^2Y and Q^2Y of pairwise OPLS-DA model.

	GDM vs. Con	n-3 Adq-GDM vs. GDM	n-3 Def-GDM vs. GDM
Liver	R^2Y:0.88, Q^2Y:0.79	R^2Y:0.94, Q^2Y:0.88	R^2Y:0.97, Q^2Y:0.93

Supplementary Table S2. Metabolites altered in liver of GDM offspring at 11 months old and modulating effects of n-3 PUFA.

Identification	RT (Min)	m/z	Change trend		
			GDM vs Con	n-3adq vs GDM	n-3def vs GDM
1-[4,9-Dihydro-2-(methylthio)-1,3-thiazino[6,5-b]indol-4-yl]-2-propanone	0.83	162.0271	↓	↑ **	-
1-Arachidonoylglycerophosphoinositol	12.22	643.2902	↑	↓ *	-
2-Hydroxy-4-oxo-5,12-heneicosadien-1-yl acetate	17.68	761.5907	↑	↓ *	-
Arginyl-Glutamic acid	9.00	616.1801	↑	↓ *	-
D-altro-D-manno-Heptose	0.83	233.0610	↓	↑ **	↑ **
Glutathione	0.83	308.0888	↓	↑ **	-
Isopetasoside	16.52	397.2217	↑	↓ *	-
LysoPC(22:6(4Z,7Z,10Z,13Z,16Z,19Z))	11.54	568.3379	↓	↑ **	↓ **
LysoPC(P-18:1(9Z))	14.08	506.3663	↑	↓ **	↓ **
MG(0:0/20:5(5Z,8Z,11Z,14Z,17Z)/0:0)	14.47	377.2693	↑	↓ **	-
N2-Fructopyranosylarginine	16.52	96.0324	↑	↓ *	↑ **
N-Ethyl trans-2-cis-6-nonadienamide	8.23	464.2876	↑	↓ *	↓ **
Nitrate	18.01	84.9775	↑	↓ **	↓ **
PC(14:0/18:0)	17.74	756.5515	↑	↓ *	-
PC(14:0/18:2(9Z,12Z))	17.45	752.5449	↑	↓ *	-
PC(14:0/20:3(5Z,8Z,11Z))	17.02	756.5533	↑	↓ **	-
PC(16:0/18:2(9Z,12Z))	13.71	780.5536	↓	↑ **	↑ *
PC(16:0/22:4(7Z,10Z,13Z,16Z))	17.97	832.5796	↑	↓ **	-
PC(16:0/22:5(7Z,10Z,13Z,16Z,19Z))	16.69	830.5665	↑	↓ **	-
PE(14:0/22:2(13Z,16Z))	16.01	744.5584	↑	↓ *	-
PE(18:3(6Z,9Z,12Z)/P-18:1(11Z))	16.64	724.5284	↑	↓ *	-
PE(20:3(5Z,8Z,11Z)/P-18:1(11Z))	16.01	769.5569	↑	↓ **	-
PE(O-16:1(1Z)/22:6(4Z,7Z,10Z,13Z,16Z,19Z))	16.71	748.5318	↓	↑ **	-
PE-NMe(18:2(9Z,12Z)/18:1(11Z))	14.04	756.5553	↑	↓ *	-
PS(22:2(13Z,16Z)/15:0)	17.78	824.5396	↑	↓ *	-
2,5-Dichloro-4-oxohex-2-enedioate	17.91	226.9504	↑	-	↓ **

2,8-Dihydroxyadenine	0.79	116.0814	↑	-	-
Adenosine monophosphate	0.83	348.0719	↑	-	-
Dihydroasparagusic acid	18.23	96.9773	↓	-	-
Hexyl benzoate	10.15	583.3291	↑	-	-
LysoPC(15:0)	14.02	482.3192	↓	-	↓ *
LysoPC(16:0)	11.95	496.3401	↑	-	-
LysoPC(18:0)	13.71	524.3702	↓	-	-
LysoPC(18:1(9Z))	12.70	522.3575	↓	-	↓ **
LysoPE(0:0/18:0)	13.67	482.3279	↓	-	-
LysoPE(0:0/20:2(11Z,14Z))	11.89	528.3044	↓	-	↓ **
LysoPE(18:0/0:0)	13.67	341.3063	↓	-	↓ *
MG(0:0/18:1(11Z)/0:0)	15.88	379.2849	↓	-	↓ **
N2-Fructopyranosylarginine	14.20	337.1706	↑	-	-
Palmitic acid	8.64	274.2722	↑	-	-
PC(18:0/18:3(9Z,12Z,15Z))	15.59	784.5858	↓	-	↑ *
PC(18:0/22:6(4Z,7Z,10Z,13Z,16Z,19Z))	17.02	856.5822	↓	-	-
PC(18:0/P-18:1(11Z))	16.01	794.6018	↑	-	-
PE(14:0/22:4(7Z,10Z,13Z,16Z))	17.04	762.5082	↓	-	-
PE(15:0/24:0)	15.97	812.6143	↓	-	-
PE(16:0/20:4(5Z,8Z,11Z,14Z))	17.04	144.9913	↓	-	↓ **
PE(16:0/22:4(7Z,10Z,13Z,16Z))	16.12	790.5354	↓	-	-
PE(20:3(5Z,8Z,11Z)/22:5(4Z,7Z,10Z,13Z,16Z))	15.04	833.5901	↓	-	-
PE(P-16:0e/0:0)	12.70	438.3024	↓	-	↓ *
PE-NMe(18:0/20:1(11Z))	14.29	810.5989	↓	-	-
PE-NMe(18:3(9Z,12Z,15Z)/20:0)	17.45	784.5837	↑	-	↑ **
PE-NMe2(18:1(9Z)/18:1(9Z))	17.20	772.5825	↑	-	↑ *
PG(16:0/18:1(11Z))	15.59	766.5679	↓	-	-
PGP(18:0/22:4(7Z,10Z,13Z,16Z))	17.80	929.5263	↑	-	-
PS(16:0/16:0)	16.21	736.5092	↓	-	↓ *
SM(d18:0/16:1(9Z))	17.74	725.5534	↑	-	↑ **

TG(8:0/i-16:0/i-17:0)	15.59	731.6100	↓	-	-
25-Acetylvulgaroside 25	11.56	479.2993	↓	↓ *	-
Araliacerebroside	16.69	732.5555	↑	↑ *	↑ *
Lysyl-Methionine	13.25	555.2979	↑	↑ **	-
Oleamide	15.63	265.2553	↓	↓ **	↓ **
PA(20:4(5Z,8Z,11Z,14Z)/24:1(15Z))	17.06	824.6194	↓	↓ *	↓ *
PC(16:0/22:6(4Z,7Z,10Z,13Z,16Z,19Z))	17.32	828.5448	↑	↑ **	↑ *
PC(DiMe(9,3)/DiMe(9,3))	15.57	833.5899	↓	↓ **	-
Tryptophyl-Proline	12.20	603.2981	↓	↓ *	-

The " ↑ " and " ↓ " arrows represent a significant increasing or decreasing trend of metabolites of GDM offspring. Green arrows show modulating effect on altered metabolites, and the red shows aggravating result. "-" means no significant change. $^{*}P<0.05$, $^{**}P<0.01$, vs GDM offspring.
